# A Good Practice Guide for Organizing the Scientific Program of International Conferences

**DOI:** 10.7759/cureus.49733

**Published:** 2023-11-30

**Authors:** Isra Aljazeeri, Arthur Lorens, Erwin Offeciers, Essam Saleh, Griet Mertens, Henryk Skarzynski, Hussain Alrand, Ilona Anderson, Joachim Mueller, Paul Van de Heyning, Piotr Henryk Skarzynski, Saad Alsaleh, Tamer Mesallam, Vincent Van Rompaey, Yassin Abdelsamad, Farid Alzhrani, Abdulrahman Hagr

**Affiliations:** 1 King Abdullah Ear Specialist Center (KAESC), College of Medicine, King Saud University Medical City (KSUMC) King Saud University, Riyadh, SAU; 2 Department of Otology, Aljaber Ophthalmology and Otolaryngology Specialized Hospital, Ministry of Health, Ahsa, SAU; 3 Department of Otology, World Hearing Center, Institute of Physiology and Pathology of Hearing, Kajetany, POL; 4 European Institute for ORL-HNS, AZ Sint-Augustinus, Antwerp, BEL; 5 Department of Otolaryngology, Alexandria University, Alexandria, EGY; 6 Department of Otology, King Abdullah Medical City, Makkah, SAU; 7 Department of Otorhinolaryngology, Head and Neck Surgery, Antwerp University Hospital, Antwerp, BEL; 8 Experimental Laboratory of Translational Neurosciences and Dento-Otolaryngology, Faculty of Medicine and Health Sciences, University of Antwerp, Antwerp, BEL; 9 Department of Teleaudiology and Screening, WorldHearing Center, Institute of Physiology and Pathology of Hearing, Warsaw, POL; 10 Heart Failure and Cardiac Rehabilitation Department, Faculty of Dental Medicine, Medical University of Warsaw, Warsaw, POL; 11 Department of Otology, Institute of Sensory Organs, Kajetany, POL; 12 Department of Otology, Center of Hearing and Speech Medincus, Kajetany, POL; 13 Otolaryngology, Ministry of Health, Dubai, ARE; 14 Department of Clinical Research, MED-EL Medical Electronics, Innsbruck, AUT; 15 Department of Otology, Ludwig Maximilian University of Munich, Munich, DEU; 16 Department of Otolaryngology - Head and Neck Surgery, College of Medicine, King Saud University Medical City (KSUMC) King Saud University, Riyadh, SAU; 17 Voice, Swallowing, and Communication Disorders Unit, Department of Otolaryngology, College of Medicine, King Saud University Medical City, Riyadh, SAU; 18 Department of Research, MED-EL GmbH, Riyadh, SAU

**Keywords:** medical education, organization, science, congress, conference

## Abstract

This paper provides a step-by-step guide for organizing the scientific program (OSP) of international conferences. Through informal discussions, a panel of experts organizing international conferences came up with this guide, which includes a flowchart, checklist, and detailed discussions of each step. Subsequently, additional specialists were invited to evaluate this synopsis and provide their input. All of the participants approved the final version after the outline was improved. This guide proposes the following six steps: 1) preparation, 2) recruitment, 3) building the agenda, 4) cross-checking the program, 5) reviewing and finalizing, and 6) in-conference refining. Thirteen items are specified across the six main steps in a detailed checklist. This OSP guide includes a flowchart and a checklist for providing a comprehensive manual for establishing, conducting, and organizing international scientific conferences. Understanding the procedures that are expected to be followed when holding a scientific conference enables the involved parties to organize and assign tasks to one another as well as create a schedule that allows them to finish their work on time. This guide can be used at any kind of scientific conference to describe an organized process, resulting in a professional and distinguished scientific program.

## Introduction

Conferences are an integral part of the scientific enterprise. “Scientific conference” has been defined as a meeting that invites submissions for presenting research results [1]. The core of today’s conference speakers presenting their work to an audience has not changed since the meetings of the Royal Society in the 1660s [2]. By the 19th century, science had started to transcend national differences and become increasingly international [3].

Gathering scientists of diverse backgrounds at conferences results in an enriching learning experience for both the audience and the participants [4]. Conferences allow scientists to present and discuss their experiences and research [5]. The unconstrained environment of a conference provides an opportunity for scientists to defend their work, engage in collegial arguments and discussions, and receive feedback, peer reviews, and critiques [6]. Brainstorming during these discussions can help develop solutions [7]. Conferences also provide the opportunity to connect, helping to create a network for collaboration and career development [5,8].

Conducting national and international conferences is becoming a routine role for scientific professionals and societies [5]. Scientists, however, are not trained for leadership roles and are often described as “accidental administrators” [9-11]. There is a lack of published literature on how to conduct such a complicated task and how to collaborate between organizations and scientists [12]. The objective of this article is to propose a good practice guide to be used for organizing scientific conferences.

## Materials and methods

Achieving a good scientific program for international conferences requires collaborative efforts between qualified professionals with extensive experience in communication, organization, and knowledge about the targeted attendees, their needs, and their interests. The execution of this task can be described in multiple orderly steps to simplify the implementation. This study tries to elaborate on the steps of organizing the scientific program of international conferences in a manner that can be used as a guide. To reach this guide, a panel of experts, in organizing international conferences through informal discussions, has outlined a flowchart and a checklist and discussed each step in detail. Further experts were then included and asked to review this outline and give their feedback. The outline was refined, and the final version was approved by all the participants.

Organizing scientific program flowcharts and checklist

**Figure 1:**
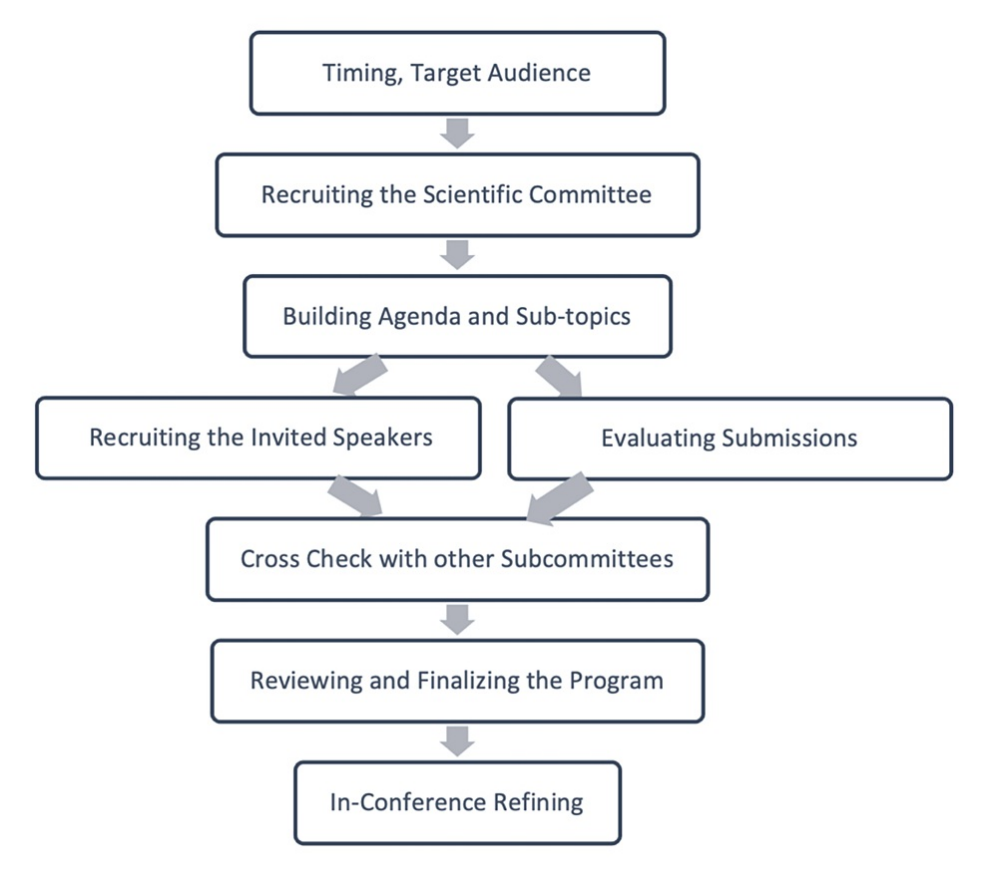
OSP (Organizing Scientific Program) Flowchart

This article provides a flowchart (Fig. 1) and a checklist (Table 1) that summarize the step-by-step process for organizing the scientific program. The text further describes the details of each step.

**Table 1:** Organizing the Scientific Program (OSP) Checklist

Item	Details	To be done by
Set a date	The conference date should be set before carrying out the process. This will help in tracking the appropriate progress and helps invitees to arrange their schedule.	The organizing committee
Recruit the main scientific committee	The main scientific committee should be leaders in the field with experience in conducting and organizing national and international conferences.	The organizing committee
Establish the vision	The vision of the conference includes who the target audience are and how long the conference will last.	The organizing and the main scientific committee
Recruit the scientific subcommittees	The main scientific committee chooses the volunteer scientific subcommittee members via CV review.	The main scientific committee
Recruit the secretaries	Each subcommittee needs a secretary whose duties last until the conference is over	The organizing committee
Organization platform	The secretaries need to build a data sheet to organize the process. This can include a sheet for the proposed program, for the confirmed program, for the submissions, and for the speakers list in which their acceptance status is documented.	The secretaries
Build the preliminary program	1-Determining the main agendas	The scientific subcommittee members
	2-Proposing detailed subtopics for each agenda	
	3-Inviting speakers	
Invitation Email	The secretaries need to sign-up new emails specific for the conference. These emails will be used to invite the speakers and receive their responses.	The secretaries
Launch the submission portal	Conference website should include a submission portal with an easy and clear submission process.	The organizing committee
Evaluate the submissions	The submissions received through the submission portal need to be blindly evaluated by multiple sub-committee members, when possible.	The scientific subcommittee members
Crosscheck with other subcommittees	Relevant subcommittees need to be in close communication to ensure that the topics of the invited speakers and the submissions are appropriated to the most suitable subcommittee.	The scientific subcommittee members
Review and finalize the program	The final program of each subcommittee needs to be first reviewed by each subcommittee and then by the main scientific committee.	The main scientific committee, the scientific subcommittees, and the secretaries
In-conference refining	To fix any last-minute changes or cancellations.	The scientific subcommittee members and their secretaries

Preparation

General Theme and Target Audience

Before starting the process of conducting a conference, there should be a clear vision of what the conference will be about: what is the aim of the conference? Who is the target audience? One of the key decisions is to give the conference a clear and attractive title. This will help give the conference its identity.

Timing

Timing of the Conference

Choosing the appropriate timing for the conference is an essential element for ensuring good attendance by both the speakers and the audience. Care must be taken to avoid national holidays and events and to avoid any other conferences with the same target audience and society.

Local tourism is one of the encouraging aspects of attendance, and choosing a season with more welcoming weather is advised.

Timing of the Preparation

Preparing a successful international scientific program requires substantial time and effort. For a large international conference, approximately more than one year of preparation time is mandatory and an acceptable duration to go stepwise through the whole process from the first advert to the final conference. As there are a large number of annual conferences, planning for the next conference should be started as early as possible after finishing the last conference. Time will be needed to promote the conference and attract the audience. The organizers need time for preparation during their workload. Dividing the preparation process into phases with specific tasks and amount of work can help the organizers fit these tasks into their daily workload. Speakers and attendees need time to schedule their travel arrangements and, thus, need to be informed about the confirmation of their participation well beforehand.

Organizing the committee

The organizing committee is responsible for the conference. This includes conducting and executing the scientific program as well as choosing a professional conference organizer (PCO) for executing the actual tasks of the organization. This includes (but is not limited to) setting the date of the conference, creating the budget, creating the website and submission portal, arranging the Continuous Medical Education (CME) certification, registering the attendees, selecting and preparing the venue including the necessary audio-visual/IT requirements and catering, finding sponsorship, and conducting marketing for the conference [13].

The organizing committee needs to take into consideration the recognizability of the conference by using the same logo for recurring editions of the same conferences.

The details of the organizing committee’s work are beyond the scope of this article.

Recruitment

Recruiting Scientific Committees

The organizing committee needs to recruit a group of committed members to prepare the scientific program. To do so, it is first needed to have a vision of the scientific background and a vision of the conference. If necessary, depending on the general content of a conference, it may be necessary to divide the scientific program into subspecialties and assign committee members to subcommittees for each subspeciality (conference in conference principle). Each subcommittee should be composed of both senior members, who play a more supervisory role, and active energetic junior members, who complete the time-consuming tasks. Being a member of a scientific committee at an international conference is a great honor and a prestigious duty.

Recruitment of the committee members should be based on merit. To resolve this issue, we propose the following process to provide a fair opportunity for all the scientific society members. An opening for the scientific committee can be advertised to society members. For medical scientific conferences, committee members should be, at minimum, a consultant with a fellowship training subspecialty. Prior participation in scientific conferences and each potential participant’s scientific CV can be objective measures in prioritizing the selection of subcommittee members. In addition, the scientific subcommittees should encourage the participation of well-known figures because their presence adds value due to their position in the scientific community.

All the members need to be active in the scientific society of their field; that is, they have attended and participated in recent conferences or have published substantially in well-known journals and should be ready and willing to work collaboratively with their fellow committee members. Commitment and enthusiasm are imperative for adhering to timelines and fulfilling responsibilities [14]. Each scientific subcommittee needs an advisory and an ad hoc member that advises the main sub-committee members. Due to their experience, the committee members of the previous scientific program can provide valuable advice. This should help to avoid foreseeable challenges. Lastly, each scientific subcommittee needs an assigned secretary.

The scientific committee should include enough members to be able to perform all tasks without adding extra members who are not performing actual tasks. Having a large number of members can also complicate decision-making due to conflicting ideas.

Setting terms of reference (TOR)

The Scientific Committee is composed of the 1) Scientific Committee Chair, 2) Deputy Chair, 3) Subcommittee Chairs, and 4) Members. Each has their own responsibilities and duties.

The Scientific Committee Chair is responsible for organizing and chairing the main scientific committee conference, including the meetings needed for the organization process. The Scientific Committee Chair will determine the agenda for each meeting, facilitating communication between the Scientific Committee and the Organizing Committee. They will be delegating responsibilities to the Deputy Chair, as appropriate.

The Deputy Chair is responsible for assisting the Scientific Committee Chair in carrying out the duties listed above. The Deputy Chair will be deputizing for the Scientific Committee Chair at meetings, as necessary.

The Subcommittee Chairs are responsible for calling a meeting with the Members regularly and delivering the minutes of the meetings to the main Scientific Committee Chair. The Subcommittee Chairs will be giving tasks to each member, setting deadlines for their completion, and raising proposals of suggestions to be discussed with the main Scientific Committee. Additionally, they will review the inputs received from the members for a final decision.

Members are responsible for completing the tasks assigned to them by their Subcommittee Chair and actively raising their concerns and recommendations to the chair for improving each step they are participating in.

Before proceeding to the details of the scientific program, the hours and number of rooms allotted in the scientific program for each subcommittee should be determined.

Building the agenda

Building the Scientific Program Agenda

After having a defined structure of the conference, including the topic, number of rooms, and duration of the conference, each subcommittee should start by setting a broad agenda for their program. The agenda topics should cover three main categories: the basics and fundamentals of the scientific topic covered in traditional routine teaching, the recent advances in the area of interest, and the controversial topics.

The agenda for the first category can be found in the table of contents of basic textbooks and targets the more junior and less experienced audience. The second and third categories can be found through extensive literature review, focusing on recent articles published in top-ranked journals. These categories target the more subspecialized, senior audience and give vision to juniors who are new in the field.

For some conference topics, it may also be helpful to include the latest industrial developments. However, it is imperative that the scientific program of a conference should be created in full independence of business interests (i.e., without interference by sponsoring companies). Companies can bring their research and development insights and progress to the audience through satellite sessions. In creating the scientific program, first arrange the main agenda then add the detailed subtopics. This should ensure that no major agenda item will be missing. The program cannot be created solely based on the speakers’ preferences and their recommendations because this can lead to repetition of the topic and a lack of focus on new advances. Instead, the agenda should cover a variety of topics and be decided upon by the scientific committee. Setting the main agenda can help determine which areas are insufficiently covered so that this may be corrected, with specific speakers in those areas.

An additional advantage is that appropriate time can be allocated to each topic on the agenda. When a topic is not filled out, a substitute subtopic of the same main agenda can be used.

Furthermore, the attendees can easily choose their topics of interest so as to arrange their schedule accordingly [8].

While arranging the program, it should be remembered that early morning and late afternoon sessions usually have lower attendance. Arranging high-impact, interesting, and interactive sessions and/or those given by key opinion leaders at these times will encourage better attendance [15]. However, the disadvantage of this arrangement can be missing out on these high-impact sessions due to the unconvincing timing.

Audiences tend to stay in the larger rooms. Thus, arranging the elite speakers to present talks in these rooms can encourage more attendance in subsequent talks given in these rooms.

Types of sessions

Sessions of the scientific conferences usually contain the categories mentioned in Table 2.

**Table 2:** Summary of the Session Types in a Scientific Program

Type of Session	Definition
Free Paper	This session presents a research work.
Keynote Lecture	It is a detailed presentation of a specific topic in a comprehensive scholastic manner by an expert. This session usually takes longer time than the free paper session.
Round Table	It contains short, individual, presentations by a number of experts on a specific topic.
Panel Discussion	An open discussion between the experts (panelists) on a specific topic is carried on with questions directed by a moderator who tries to share all the options and different aspects of decision-making.
Debate	Two or more different or opposing opinions on the same topic are debated in which each expert illustrates the pros and cons of his opinion.

Definition

Free paper: This session presents a research work.

Keynote lecture: It is a detailed presentation of a specific topic in a comprehensive scholastic manner by an expert. This session usually takes longer time than the free paper session.

Round table: It contains short, individual, presentations by a number of experts on a specific topic.

Panel discussion: An open discussion between the experts (panelists) on a specific topic is carried on with questions directed by a moderator who tries to share all the options and different aspects of decision-making.

Debate: Two or more different or opposing opinions on the same topic are debated in which each expert illustrates the pros and cons of his opinion.

Types of streaming

In the recent era of remote communication, conferences are not bound by physical presence. Streaming can be physical, digital via a video conference, or hybrid.

The digital type can be either live streaming or recorded videos that are available for the audience for a period of time.

In this article, we are most focused on in-person, physical conferences.

Recruiting the speakers

The speakers are divided into two categories: invited speakers and speakers who submitted their work through the submission portal. The scientific committee needs to decide which of these two categories they are going to base their program on. This decision is also influenced by the session formats, which are to be used in the conference and covered by key persons. However, key persons are needed as lighthouses to promote the conference. This decision will define the next steps.

There are benefits to having the invited speakers make up the majority of the scientific program. This can help in having control over the quality of the program. The lengthy portions of the program such as the keynote presentations, panel discussions, and instructional courses need carefully selected and well-known speakers. The scientific committee can control who these speakers are, if they are chosen by invitation instead of a more passive submission portal candidate. Moreover, personal invitations can lead to participation in cases where speakers do not know about the conference or forget to submit their work. Inviting elite speakers should increase the likelihood they present and this, in turn, could attract a greater number of conference attendees.

Distinguished speakers to invite can be identified through the following pathways: First, an extensive literature review can identify authors with numerous publications on highly specialized topics [16]. These publications need to be recent and published in high-ranked journals. The second pathway is that the representatives of the national societies are generally well-known figures in their area. By searching for national representatives, the program can involve equity in the distribution of the speakers. In most cases, the elite and distinguished speakers are usually well-known to the scientific society. Additionally, the attendees of previous conferences can have a very beneficial input on the recent speakers who are interested in participating in international conferences and those who are admired by the attendees. One can go through the scientific program of any recent international conferences, which are a good guide to finding active speakers and hot topics. Contact search in researcher-oriented websites such as ResearchGate can also lead to finding high-ranked researchers and their contacts.

Continual follow-up (monitoring and plotting the progress)

Scientific subcommittee members should hold regularly scheduled meetings, according to the workload, to follow up on the progress.

There is a need to follow how many hours are confirmed by the speakers, track the number of submissions, and implement the confirmed speakers’ suggestions into the agendas. This will reveal the shortcomings in each agenda area. Scientific subcommittee members can then direct the invitation process to build a more comprehensive program; for example, they plan the steps of building the scientific program.

This task is accomplished by revising the recruitment process through the previously mentioned pathway to ensure that all agendas are complete and that there is a fair global contribution by the speakers.

Some speakers may accept the invitation without providing their topics. If this occurs, follow-up emails will need to be sent.

It is also possible to send tailored invitations to highly specialized speakers to present on topics/areas that are missing or underrepresented in the scientific program. Using the pathways mentioned, potential speakers are more likely to accept invitations to speak on subtopics they are familiar with and enthusiastic about.

The main Scientific Committee will receive the weekly report about the progress of each subcommittee to plot the difference between each subcommittee and create more engagement.

Evaluating the submitted abstracts through the submission portal

The submission portal needs to be implemented into the conference website in the early phase of the process to allow enough time for possible participants to know about the conference and prepare and submit their work. The rules and regulations for submission need to be stated clearly and simply. The submission portal needs to be smoothly accessible through the website and through the links that are distributed to the community.

A primary deadline for submission needs to be set from the beginning. By setting a primary deadline, potential participants will be encouraged to submit their work early, which will allow the scientific committee to know what action they need to take. If the submitted manuscripts are of adequate quality and length to fill in the gaps that are not covered by the invited speakers, extending the deadline will not be necessary.

Cross-checking the program

Cross-checking with Other Subcommittees

It is not uncommon that some agendas of subcommittees overlap. The invited speakers may respond to one subcommittee’s invitation, but their suggested topics fit another subcommittee better. The same issue may occur with submitted manuscripts through the submission portal. Subcommittees, therefore, need to be in close communication so that they can transfer the submitted work to the most suitable subcommittee.

This can be made by a program planning software for automated checkups. However, it is also important to have a manual checkup to ensure there is nothing missed by the software.

Promoting the conference

To encourage submissions and attendance to the program, the committee should announce the conference at an early stage and according to the time course left to the conference dates in a proper way (first announcement with dates and venue, second announcement with topics, and subsequently more information when the conference date comes closer, preparing early a preliminary program and general outline). This program should consist of interesting and attractive topics presented by distinguished speakers whose participation is confirmed. The preliminary program should then be distributed for advertisement by the organizing committee that handles social promotions and by the PCO for administrative issues.

Reviewing and finalizing the program

To ensure that there is no human error or typos, the final program should be reviewed in multiple rounds by more than one person. Each subcommittee’s final program should be first reviewed by each subcommittee and then by the main Scientific Committee as follows:

The subcommittee ensures that, either through email or the submission portal, every subtopic in the program is based on a documented speaker submission.

The subcommittee ensures that every speaker’s slot in the program is as appropriate to their significance.

The main Scientific Committee ensures that there is no repetition in the program across the various subcommittees and that each subtopic is handled by the most appropriate subcommittee.

The main Scientific Committee ensures that speakers are not scheduled for different rooms at the same time.

Formally, the final program should be printed, and the schedule should be prepared carefully to allow a quick overview.

In-conference refining

The speakers in the program can make numerous requests for last-minute changes or cancellations. This needs to be addressed by the scientific subcommittee members, as per their judgment and knowledge of the program and relations to other speakers. A good rapport can be of critical value in these situations.

Subcommittee members and their secretaries must be easily accessible and follow their communication portals regularly. There should also be close communication with the organizing committee to ensure speakers’ attendance beforehand to be able to plan any changes in advance.

Interactivity During Sessions

One of the vital sections of each session is the questions asked by the attendees, which opens the door for interaction and a deeper understanding of the material presented. New technologies such as using applications that allow the audience to write down their questions can help a lot in easier and more organized questioning. The time allowed for the questions must be pre-defined by the scientific committee while making the program schedule and be maintained by the session moderators to avoid being behind schedule.

## Results

The panel of experts included five participants who conducted an open-ended discussion on the details of conducting and organizing the scientific program of international conferences. An extensive literature review revealed the paucity of published material discussing the same process in detail. The panel finally decided on six fundamental steps and a 13-item checklist. These steps and items were then sent to an additional 12 experts, who critically reviewed and revised these steps and items and provided their feedback. These feedbacks were implemented on the primary steps and checklist, and the final version was formulated, as illustrated in the next section. The final version was approved by all the experts. The panel also decided on a flowchart summarizing the steps needed to be taken by the scientific committee to establish the scientific program (Figure 1).

## Discussion

In this article, we introduce a good practice guide for OSP of international conferences. Developing a good practice guide is a complex process that requires the cooperation of a panel of experts. First, the need for developing the guide was identified and justified. Then, the literature was reviewed to identify similar work and the evidence needed to make the guide. Rigor must be applied to assessing the published studies to utilize any previous efforts. However, the current literature was found to be limited in describing the process of OSP [17,18].

The use of expert opinion has been justified by Warsh [19], as the primary basis for a guideline when scientific evidence is weak or nonexistent, which is what we have faced in developing this guide due to the lack of proper evidence after a thorough literature review. When a guide is written by experts, it has the advantage of illustrating the process that has already been applied and proven to be effective.

Warsh defined the principles of developing a good practice guide as having clarity in the definitions, compatibility of each attribute and its definition with professional usage, clear rationales or justifications, and sensitivity to practical issues. Any guide needs to be practical and informative. The instructions should be feasible and replicable. These principles were considered in developing this guide [19].

## Conclusions

Organizers of international scientific programs need to have a team of dedicated, knowledgeable experts. We believe that scientific programs of the conferences need to be conducted in an organized manner. This guide summarizes the collective experience of the authors, with their diverse backgrounds and long history of conducting numerous international conferences. This guide also provides a practical, detailed, and step-by-step process for conducting and organizing international scientific conferences. Knowing the steps anticipated in conducting a scientific conference helps the participating parties plan and divide the workload among themselves and prepare a timely schedule for accomplishing their tasks. Additionally, this guide could be applied to any type of scientific conference. The definitions used in this guide help in assigning the appropriate person for each task, while the flowchart and the checklist illustrated in this guide can help in evaluating the progress of the organization process.
